# First Report of *Rickettsia conorii* in *Hyalomma kumari* Ticks

**DOI:** 10.3390/ani13091488

**Published:** 2023-04-27

**Authors:** Shafi Ullah, Abdulaziz Alouffi, Mashal M. Almutairi, Nabila Islam, Gauhar Rehman, Zia Ul Islam, Haroon Ahmed, Itabajara da Silva Vaz Júnior, Marcelo B. Labruna, Tetsuya Tanaka, Abid Ali

**Affiliations:** 1 Department of Zoology, Abdul Wali Khan University Mardan, Mardan 23200, Pakistan; 2 King Abdulaziz City for Science and Technology, Riyadh 12354, Saudi Arabia; 3 Department of Pharmacology and Toxicology, College of Pharmacy, King Saud University, Riyadh 11451, Saudi Arabia; 4 Department of Chemistry, Abdul Wali Khan University Mardan, Mardan 23200, Pakistan; 5 Department of Biotechnology, Abdul Wali Khan University Mardan, Mardan 23200, Pakistan; 6 Department of Biosciences, COMSATS University Islamabad (CUI), Islamabad 44000, Pakistan; 7 Centro de Biotecnologia and Faculdade de Veterinária, Universidade Federal do Rio Grande do Sul, Porto Alegre 91501-970, Brazil; 8 Department of Preventive Veterinary Medicine and Animal Health, Faculty of Veterinary Medicine, University of São Paulo, Sao Paulo 05508-060, Brazil; 9 Laboratory of Infectious Diseases, Joint Faculty of Veterinary Medicine, Kagoshima University, Kagoshima 890-0065, Japan

**Keywords:** *Hyalomma kumari*, Ixodidae, small ruminants, *Rickettsia conorii*, Pakistan

## Abstract

**Simple Summary:**

Ticks are blood-feeding ectoparasites that transmit life-threatening pathogens to humans and animals. Only 10% of all identified tick species have been screened for different tick-borne pathogens. *Hyalomma* ticks are associated with a wide range of pathogens including *Theileria* species, *Babesia* species, *Anaplasma* species, *Ehrlichia* species, and *Rickettsia* species. Moreover, ticks of genus *Hyalomma* are vectors for the Crimean-Congo hemorrhagic fever (CCHF), a serious threat endemic in Pakistan. In Pakistan, different tick species have been found positive for rickettsial agents; however, *Hyalomma kumari* ticks have never been investigated for any potential pathogens. In this work, *H. kumari* ticks were collected from goats and sheep, and morphologically and molecularly identified using different genetic markers. The identified ticks were screened for rickettsial agents using genetic markers that resulted in the detection of *Rickettsia conorii* for the first time in this tick. A proper surveillance program should be designed to effectively avoid any zoonotic consequences associated with these rickettsial pathogens.

**Abstract:**

As a vector of wide range of pathogenic agents, ticks pose health threats to wild and domestic animals, and humans. Information is unavailable about the prevalence and spatial survey of *Hyalomma kumari* ticks and associated *Rickettsia* spp. in Pakistan. Concerning this knowledge gap, the present study aimed to molecularly detect *Rickettsia* species associated with *H. kumari* infesting small ruminants in Khyber Pakhtunkhwa (KP), Pakistan. A total of 409 *H. kumari* ticks were collected from 163/295 infested hosts with an infestation rate of 55.25%. A total of 204 females, 158 males, and 47 nymphs were collected. Goats were heavily infested by 224 ticks having an infestation rate of 58.33% (98/168), whereas sheep were infested by 185 ticks having a lesser infestation rate of 51.18% (65/127). Genomic DNA extracted from ticks was used for the amplification of tick (*cox I*, 16S rRNA, ITS-2) species and *Rickettsia* (*gltA*, *ompA*, and *ompB*) partial genes. Eighty-three ticks were subjected to PCR, and 8/83 (9.6%) were found positive for rickettsial agents. The *cox I* and 16S rRNA sequences of *H. kumari* showed 98.90–99.74% identity with *H. kumari* sequences reported from Pakistan, and phylogenetically clustered to the corresponding species reported from Pakistan and India. The obtained rickettsial *gltA*, *ompA*, and *ompB* sequences showed 100% identity with *Rickettsia* sp. of the *Rickettsia conorii* reported from Pakistan. In the phylogenetic trees, rickettsial sequences clustered with uncharacterized *Rickettsia* sp. from Pakistan and *R. conorii* from Israel, Russia, South Africa, and India. The present molecular based detection of *H. kumari*-associated *R. conorii* will facilitate effective surveillance in the region.

## 1. Introduction

In the tropical and subtropical climates, the ixodid ticks of the genus *Hyalomma* are of great medical and veterinary importance in terms of health risks and economic burden [1,2]. *Hyalomma* ticks are associated with a wide range of pathogens including *Theileria* spp. [3,4], *Babesia* spp. [5], *Anaplasma* spp., *Ehrlichia* spp. [6], and *Rickettsia* spp. [7]. Moreover, ticks of genus *Hyalomma* are vectors for the Crimean-Congo hemorrhagic fever (CCHF), a serious threat endemic in Pakistan [8]. To date, 27 species of *Hyalomma* have been identified in Palearctic, Oriental, and African regions [9], of which 13 have been reported in Pakistan [2,10,11,12,13,14,15]. The genus *Hyalomma* has been classified into two subgenera, i.e., *Euhyalomma* and *Hyalommina*. The most diversified subgenus *Euhyalomma* is abundant in Africa, Asia, and Europe while species of subgenus *Hyalommina* are divided in to two groups based on specificity to their geographies. *Hyalomma punt*, *Hyalomma rhipicephaloides,* and *Hyalomma* arabica have been distributed in the Arabian Peninsula, northeast Africa, and the Near East, while *Hyalomma hussaini*, *Hyalomma brevipunctata,* and *Hyalomma kumari* are restricted to South Asia [16].

Subtropical and harsh semi-arid climatic conditions are ideal for the development and growth of many *Hyalomma* species [17,18]. Located in subtropics, the majority of the rural population in Pakistan relies on livestock, particularly small ruminants, for dairy and meat products. Pakistan has a huge population of small ruminants that consists of approximately 78.2 million goats and 31.2 million sheep [19]. The list of *Hyalomma* ticks infesting small ruminants in Pakistan includes *Hyalomma anatolicum*, *Hyalomma dromedarii*, *Hyalomma isaaci*, *Hyalomma hussaini*, *Hyalomma scupense*, *Hyalomma asiaticum*, *Hyalomma kumari,* and *Hyalomma turanicum* [2,12,13,20,21,22].

The Rickettsiaceae represents a broad group of Gram-negative intracellular bacteria that can serve as symbionts or pathogens and infect a broad range of hosts. The genus *Rickettsia*, which was first recognized as the agents responsible for spotted fever and other rickettsioses in vertebrate hosts, which spread through ticks, lice, fleas, and mites, is perhaps the best-known group of Rickettsiaceae [23]. Globally, *Rickettsia conorii* has been detected in various species including *Rhipicephalus sanguineus*, *Rhipicephalus turanicus, Rhipicephalus bursa, Rhipicephalus pumilio, Rhipicephalus evertsi, Rhipicephalus simus, Rhipicephalus mushamae, Haemaphysalis leechi,* and *Haemaphysalis punctaleechi* in several countries such as France, Bulgaria, Turkey, India, African, and Caspian countries [24]. So far, 25 species of *Rickettsia* are known to have pathogenic potential causing rickettsioses, including spotted fever group (SFG) rickettsioses, as for example, Mediterranean spotted fever (*Rickettsia conorii conorii*) in Europe and Africa, African tick bite fever (*Rickettsia africae*) in Africa, and Rocky Mountain spotted fever (*Rickettsia rickettsii*) in the Americas [25]. In addition, *Rickettsia conorii israelensis* is the causative agent of Israeli spotted fever in the Mediterranean region, *Rickettsia conorii caspia* is the etiological agent of Astrakhan fever in Caspian Sea region, northern Africa, and some Mediterranean countries, and *Rickettsia conorii indica* is the agent of Indian tick typhus in the Oriental region and southern Europe [26,27]. In Pakistan, there is a single earlier record of *R. conorii* in ticks using a toxin neutralization test (TNT) [28].

The advancement of molecular approaches as well as the increasing number of tick sequences annotated in GenBank enable a complementary categorization strategy based on genetic characteristics rather than only morphology [29,30,31]. In recent years, this methodology was adopted to identify and classify various tick groups, allowing for a re-definition of species distribution as well as detailed studies on tick-host preferences and population dynamics [32,33]. Mitochondrial genes are helpful as genetic markers because of the strict maternal inheritance and in some conditions, a quicker evolutionary change rate [34,35]. These markers have been used to classify and investigate tick populations, resulting in a huge amount of data on tick mitochondrial markers and improved field sample classification [36]. This tick has never been reported for rickettsial agent, although several other tick species have been observed as positive for different *Rickettsia* spp. in the region. The molecular and phylogenetic characterization of *Hyalomma* ticks, especially *H. kumari* and their associated *Rickettsia*, has been neglected in Pakistan. Keeping in view, this study was focused on investigating the molecular surveillance of rickettsial agents associated with *H. kumari* parasitizing small ruminants in Pakistan.

## 2. Results

**Figure 1:**
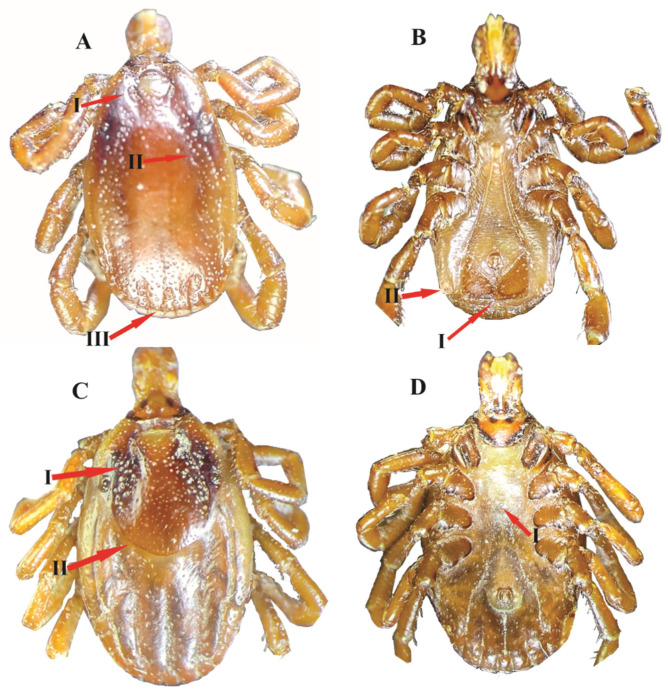
*Hyalomma kumari*, (**A**) male dorsal (I: cervical groove, II: lateral side punctation, III: posteromedian groove); (**B**) male ventral (I: adanal plates, II: accessory anal plate); (**C**) female dorsal (I: cervical field, II: scutum shape); (**D**) female ventral (I: genital aperture).

Morphologically, males of *H. kumari* were identified by two pairs (adanal and accessory adanal) of anal plates which differentiate them from the rest of *Hyalomma* (three pairs of anal plates) species. Conscutum is yellowish brown in colour with medium and small sized punctations, distributed mostly on anterior, lateral, and caudal fields. The females of *H. kumari* were identified by the yellowish brown colour scutum, as in males. The scutum is slightly longer than its breadth, with a small size, and moderately sparse punctations. The genital aperture is narrow and U-shaped (Figure 1).

### 2.1. Hosts and Spatial Survey of Ticks

Overall, 409 *H. kumari* ticks were collected from 163 infested out of 295 examined hosts with a prevalence rate of 55.25%. A total of 204 females, 158 males, and 47 nymphs were collected (Table 1). Goats were highly infested, with an infestation rate of 98/168 (58.33%) counting for 224 ticks, while sheep were less infested with a rate of 65/127 (51.18%) and counting for 185 ticks. Gender-wise, data showed a high prevalence of ticks on female goats (126), followed by female sheep (104), male goats (98), and male sheep (81) (Table 1). *Hyalomma kumari* ticks were collected from the four districts (Nowshera, Mardan, Shangla, and Mohmand) of northern Pakistan. Among the selected districts, the highest number of ticks was collected in Mohmand, 120 (57 females, 49 males, 14 nymphs), followed by Nowshera, 118 (58 females, 44 males, 16 nymphs), Shangla, 91 (48 females, 32 males, 11 nymphs), and Mardan, 80 (41 females, 33 males, 6 nymphs).

### 2.2. Sequence and Phylogenetic Analyses of Tick

**Figure 2:**
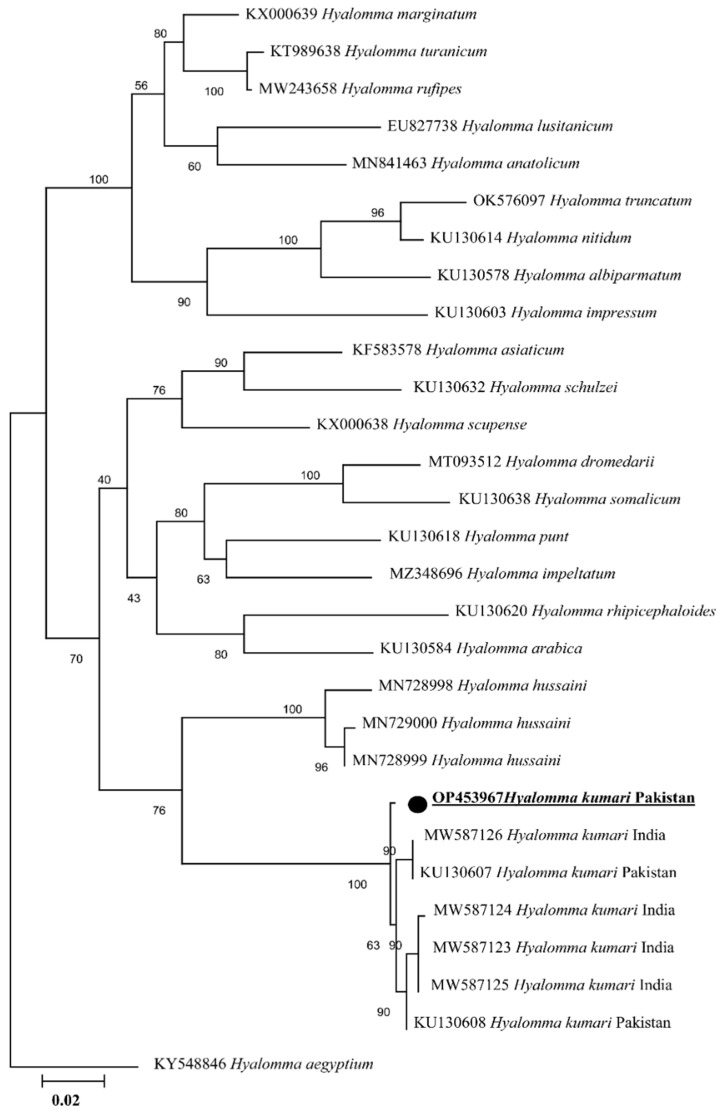
Phylogenic tree based on *cox I* partial sequences of *Hyalomma* spp. using maximum likelihood method. *Hyalomma aegyptium* (KY548846) was used as an outgroup, using 1000 bootstrap value at each node. The number of substitutions per site has been shown by scale bar. The present sequence of *H. kumari* from Pakistan is highlighted by bold font and marked with black circle.

**Figure 3:**
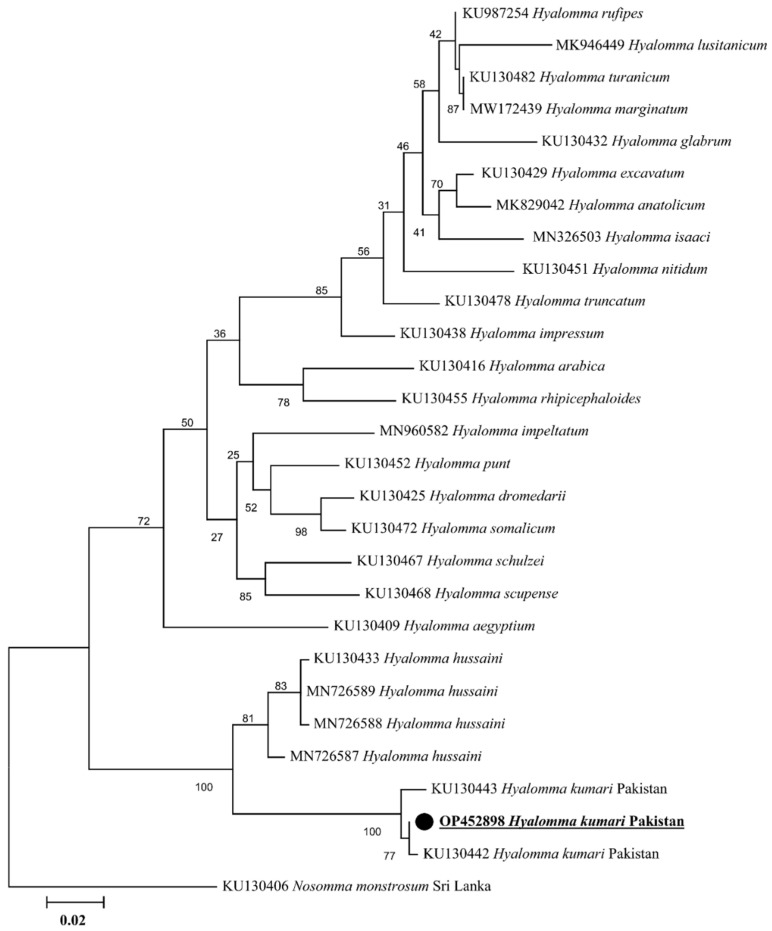
Phylogenic tree based on 16S rRNA gene partial sequences of *Hyalomma* spp. using maximum likelihood method. *Nosomma monstrosum* (KU130406) was used as an outgroup, using 1000 bootstrap value at each node. The number of substitutions per site has been shown by scale bar. The present sequence of *H. kumari* from Pakistan is highlighted by bold font and marked with black circle.

The PCR amplified *cox I*, 16S rRNA, and ITS-2 products have been shown in Supplementary Files (Figures S1–S3), respectively. The obtained identical sequences for each *cox I*, 16S rRNA, and ITS-2 fragment were considered as a single consensus sequence. In NCBI BLAST, the obtained trimmed *cox I* (755 bp) showed 98.90–99.47% identity with *H. kumari* sequences reported from Pakistan (KU130608 and KU130607) and India (MW587126, MW587125, MW587123, and MW587124). The phylogenetic tree was designed by downloading 28 sequences of various *Hyalomma* species. The obtained sequence was clustered with the sequences reported from Pakistan and India (Figure 2). The obtained *cox I* sequence was uploaded to the GenBank under accession number OP453967. The ITS-2 (778 pb) partial sequence was uploaded to the GenBank under the accession number OP454037. Similarly, the obtained trimmed 16S rRNA gene fragment (384 bp) showed 98.96–99.74% identity with *H. kumari* sequences reported from Pakistan (KU130442 and KU130443). Twenty-six sequences of different *Hyalomma* species were downloaded to design a phylogenetic tree, taking *Nosomma monstrosum* as an out group. In the phylogenetic tree, the obtained sequence clustered with *H. kumari* from Pakistan (Figure 3). Our obtained 16S rRNA gene partial sequence was uploaded to the GenBank under accession number OP452898. Due to insufficient ITS-2 sequences of *Hyalomma* species, we opted to not construct the ITS-2 based phylogenetic tree.

### 2.3. Rickettsia spp. Prevalence and Phylogenetic Analyses

Among the collected ticks, 83 samples were subjected to PCR (Table 1) for the characterization of *Rickettsia* using rickettsial primers (Table 2). The overall detection rate of *Rickettsia* was 9.6% (8/83), being highest in Nowshera with 12.5% (3/24), followed by Mardan 10.5% (2/19), Mohmand 9.5% (2/22), and Shangla 5.5% (1/18). The spatial survey of *Rickettsia* in each district is shown in Table 2.

**Figure 4:**
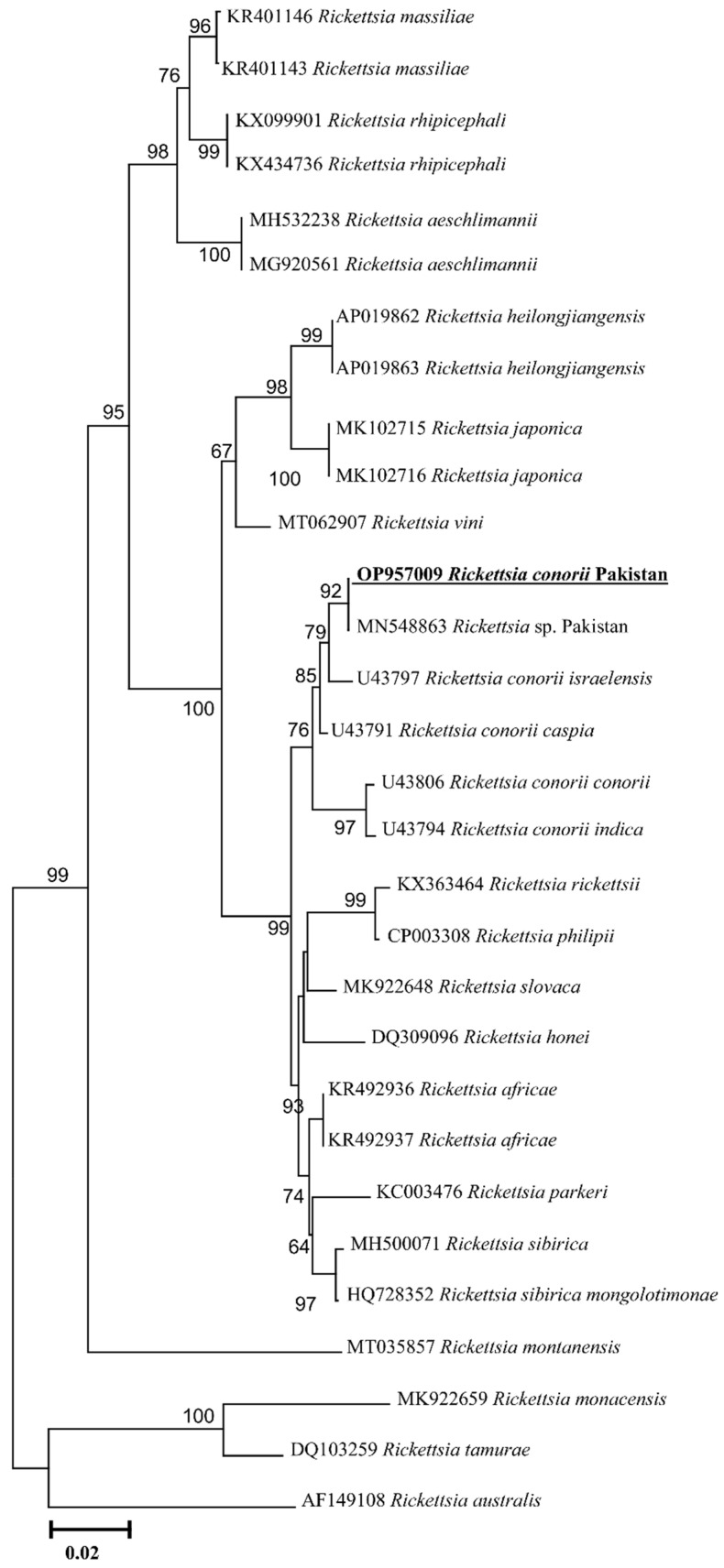
Phylogenic tree based on *ompA* partial sequences of *Rickettsia* spp. using maximum likelihood method. *Rickettsia australis* (AF149108) was used as an outgroup, using 1000 bootstrap value at each node. The number of substitutions per site has been shown by scale bar. The present sequence of *R. conorii* (OP957009) from Pakistan is highlighted by bold and underline font.

**Figure 5:**
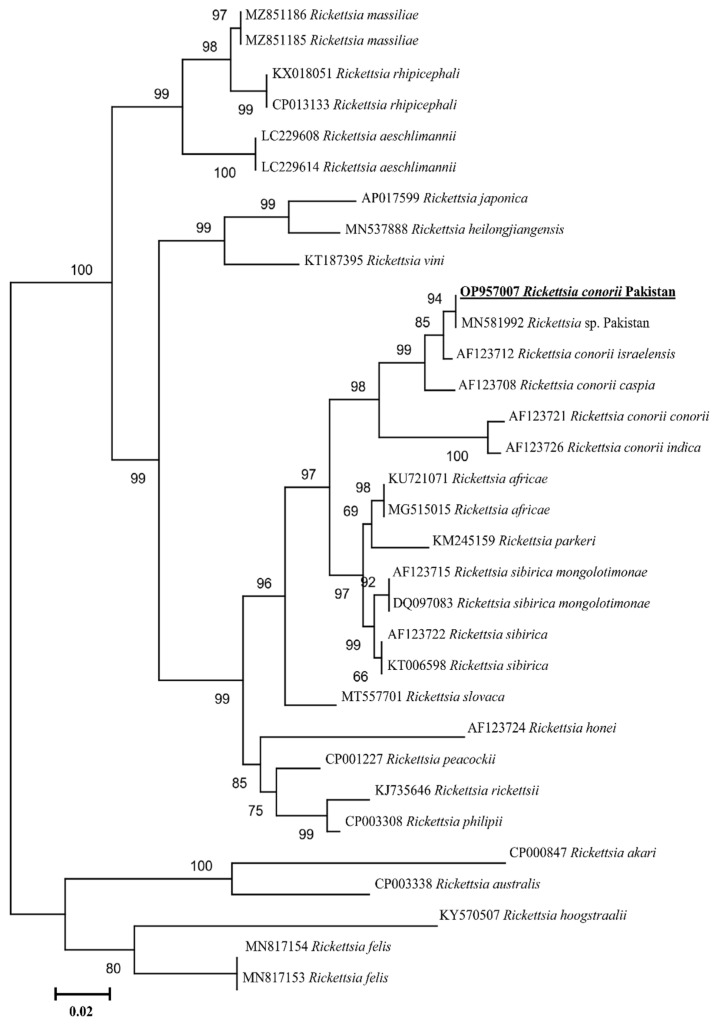
Phylogenic tree based on *ompB* sequences of *Rickettsia* spp. using maximum likelihood method. *Rickettsia felis* (MN817154, MN817153) was used as an outgroup, using 1000 bootstrap value at each node. The number of substitutions per site has been shown by scale bar. The present sequence of *R. conorii* (OP957007) from Pakistan is highlighted by bold font and marked by an underline.

**Figure 6:**
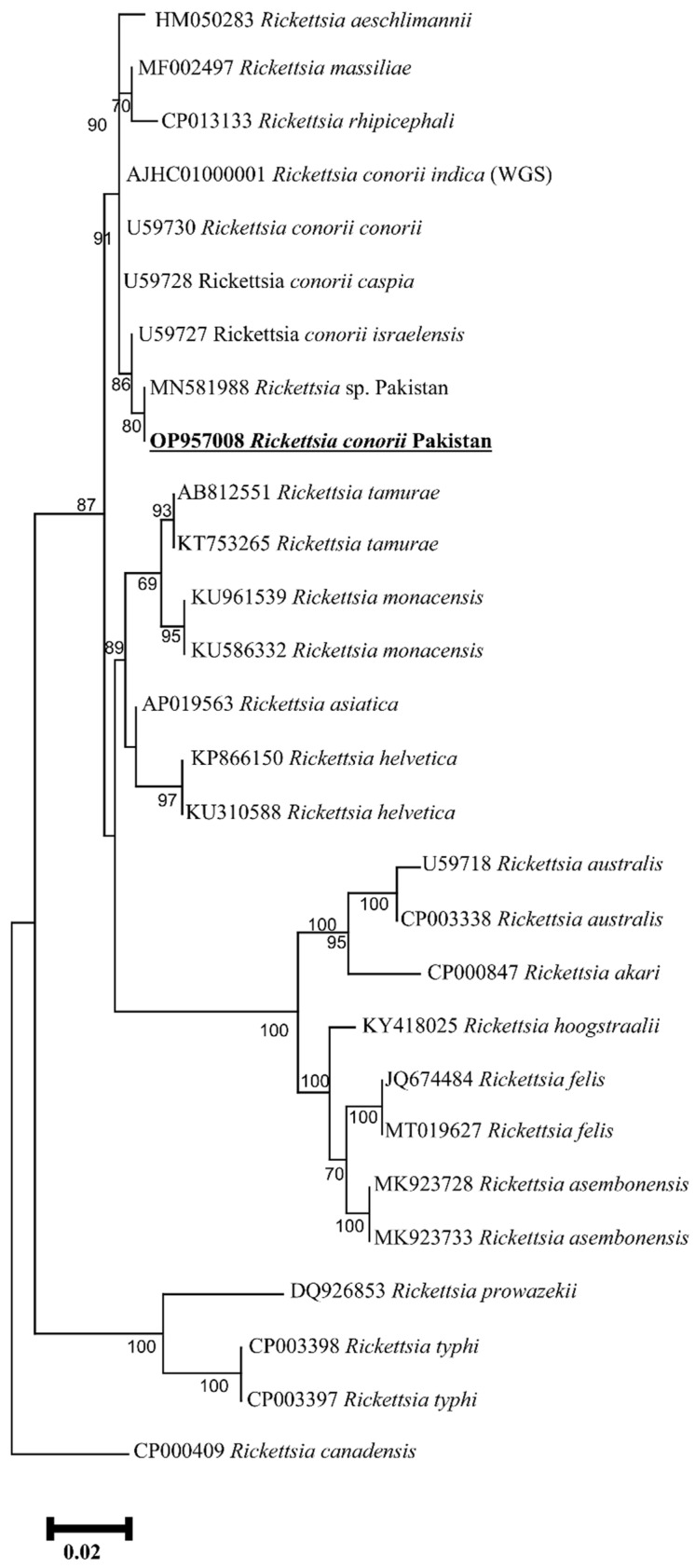
Phylogenic tree based on *gltA* partial sequences of *Rickettsia* spp. using maximum likelihood method. *Rickettsia canadensis* (CP000409) was used as an outgroup, using 1000 bootstrap value at each node. The number of substitutions per site has been shown by scale bar. The present sequence of *R. conorii* (OP957008) from Pakistan is highlighted by bold font and marked by an underline.

The PCR amplified *ompA*, *ompB*, *gltA* products have been shown in Supplementary Files (Figures S4–S6), respectively. The obtained *Rickettsia* DNA partial sequences for each gene (*ompA*, *ompB*, *gltA*) were assembled and considered as a single consensus sequence. The BLAST result for the *ompA* (466 bp) showed 100% identity with the reported sequence of the *Rickettsia* sp. From Pakistan (MN548863), 99.60% with *R. conorii israelensis* from Israel (U43797), 99.36% with *R. conorii caspia* from Russia (U43791), 97.01% with *R. conorii indica* from India (U43794), and 96.90% identity with *R. conorii conorii* from South Africa (U43806). The phylogenetic tree was designed based on the *ompA* gene by downloading 29 *ompA* sequences of different *Rickettsia* species, including all four subspecies of *R. conorii*. The obtained *ompA* sequence in the phylogenetic tree clustered with *Rickettsia* sp. From Pakistan, while grouped in a sister clade with *R. conorii israelensis* from Israel among all subspecies of the *R. conorii* (Figure 4). Our *ompA* sequence has been uploaded to the GenBank under the accession number (OP957009). Similarly, the obtained trimmed *ompB* (773 bp) partial sequence showed 100% identity with the *Rickettsia* sp. Reported from Pakistan (MN581992), 99.61% with *R. conorii israelensis* from Israel (AF123712), 99.36% with *R. conorii caspia* from Russia (AF123708), 96.64% with *R. conorii indica* from India (AF123726), and 96.51% with *R. conorii conorii* from South Africa (AF123721). The phylogenetic tree was designed by downloading 32 sequences of various *Rickettsia* species along with subspecies of *R. conorii.* The obtained sequence clustered with the *Rickettsia* sp. reported from Pakistan and *R. conorii israelensis* from Israel appeared on the sister clade (Figure 5). Our obtained *ompB* sequence was uploaded to the GenBank under accession number (OP957007). The trimmed *gltA* partial sequence (359 bp) showed 100% identity with the *Rickettsia* sp. reported from Pakistan (MN581988), 99.72% with *R. conorii israelensis* from Israel (U59727), 99.44% with *R. conorii caspia* from Russia (U59728), and 99.44% identity with *R. conorii conorii* from South Africa (U59730). The phylogenetic tree was designed by downloading 27 sequences of various *Rickettsia* species as well as subspecies of *R. conorii*. The obtained sequence was clustered with the sequences reported from Pakistan while grouped in a sister clade with *R. conorii israelensis* from Israel, among the subspecies of *R. conorii* (Figure 6). Our obtained *gltA* sequence was uploaded to the GenBank under accession number (OP957008).

## 3. Discussion

The significance of *Hyalomma* ticks as potential vectors for *Rickettsia* is under debate. Several studies have revealed the detection of different *Rickettsia* species in *Hyalomma* ticks. A limited number of studies have been conducted on the prevalence and spatial distribution of *Hyalomma* ticks, specifically *H. kumari* in Pakistan [12]. To the best of our understanding, this is the first survey investigating the spatial distribution and host-wise prevalence of *H. kumari* infesting small ruminants. The knowledge regarding the detection of *Rickettsia* spp. in *H. kumari* infesting small ruminants is inadequate. This study contributes to the neglected knowledge about *R. conorii* in *H. kumari* ticks in Pakistan.

Within a specific zoogeographic region, the prevalence and distribution of ticks have been influenced by climatic and environmental factors [37,38], with *Hyalomma* species as the top successful flourisher of harsh desert climatic conditions [9,16]. Unlike other *Hyalomma* species, *H. kumari* favors areas receiving sufficient rainfall (greater than desert regions) [12,39], as the current study was done in a region having prescribed climatic and metrological conditions. *Hyalomma kumari* is a three-host tick that infests nearly all types of domestic and some wild hosts [16]. Herein we report *H. kumari* collected from goats and sheep inhabiting less elevated hill stations in the selected Pakistan Districts, as this tick species prefers such topography [12,16]. *Rhipicephalus turanicus* and *Rhipicephalus haemaphysaloides* have been reported infesting goats and sheep in the current study region [30]. However, these two tick species were only found positive for *Rickettsia massiliae* [40,41]. The fact that no rickettsial DNA was previously detected in *H. kumari* ticks [42], motivated our work to search for *H. kumari*-associated *Rickettsia* species.

For taxonomic purposes and to identify the vectors of a wide range of pathogens, accurate classification of related tick species is essential [9]. Validating the morphological identification, recent investigations have been focused on the taxonomy and evolutionary history of ticks and tick-borne *Rickettsia* species utilizing different genetic markers [24,43,44,45]. Understanding phylogenetic relationships at the species level, in several studies the mitochondrial genes 16S rRNA and *cox I* have been implemented as appropriate genetic markers [36,45,46,47]. In 16S rRNA and *cox I* based phylogenetic trees, the *H. kumari* of the current study clustered with the same species reported from Pakistan and India. In contrast to lower identities with other countries, the Indian sequences had the highest nucleotide identity and a similar phylogeny, indicating a high genetic conservation among *H. kumari* from Pakistan and India. Before the political separation in 1947, both countries were part of the same Indo-Pak subcontinent, where animals were moving freely. The transmigration of tick-infested hosts across the borders and similar agro-ecological conditions are the reasons for such situations [48]. The grouping of *H. kumari* as a sister clade to *H. hussaini* coincides with the morphological similarities between *H. kumari* and *H. hussaini,* as both belong to the *H. kumari* group of the subgenus *Hyalommina* [16].

*Rickettsia* species are mostly zoonotic pathogens that are harbored by a wide range of arthropod vectors, especially ticks [27]. The diagnosis of *Rickettsia* in ticks is of great importance, not only to identify the infected ticks, but also to determine the risk of transmission to humans [49,50]. The occurrence of SFG *Rickettsia* is common in *Hyalomma* ticks globally [23,51], though in Pakistan, SFG *Rickettsia* have been detected in *Rhipicephalus turanicus* and *Rhipicephalus haemaphysaloides* [40,41]. To evaluate the taxonomy and phylogeny of *Rickettsia* spp., the *gltA*, *ompA*, and *ompB* genes are among the most suitable genetic markers [52]. All four subspecies of *R. conorii* (*R. conorii israelensis*, *R. conorii caspia*, *R. conorii conorii*, *R. conorii indica)* were analyzed in the phylogenetic trees based on *ompA, ompB,* and *gltA* [53]; however, the absence of a specific *gltA* sequence of *R. conorii indica* in the GenBank compelled us to include the *gltA* based aligned sequence from the whole genome shotgun (WGS) sequence of the *R. conorii indica* [54]. Herein, we detected *R. conorii* that was associated with *H. kumari* collected from goats and sheep, which validates and confirms the previous knowledge on TNT based detection of *R. conorii* in Pakistan [28]. The obtained sequences based on the three rickettsial genes (*gltA*, *ompA*, and *ompB*) showed 100% identity with unpublished *Rickettsia* sp. detected in *Rhipicephalus turanicus* collected in 1963 from goats in Pakistan [28], which may be due to the expansion of *Rickettsia* associated with tick vectors infesting migrant hosts [25]. All the *Rickettsia* phylogenetic trees clustered with reported sequences of Pakistan and formed a sister clade with *R. conorii israelensis,* revealing the evolutionary closeness.

The presence of *R. conorii* in ticks feeding on sheep and goats represents a zoonotic concern. Notably, sheep and goats are among the commonly kept animals in Pakistan [15,22] because of their high fertility, short generation period, tolerance to severe climatic conditions, and status as a source of income for rural households [55]. They are typically kept as free-ranging animals and are frequently tethered at night close to human settlements. This creates a favorable condition for both interactions with disease carriers such as ticks, enabling the spread of pathogens like *R. conorii* to humans. Further studies should evaluate the transmission of *R. conorii israelensis* by these ticks to humans and animals, to understand any zoonotic consequences related to these parasites.

## 4. Materials and Methods

### 4.1. Study Area and Tick Collection

**Figure 7:**
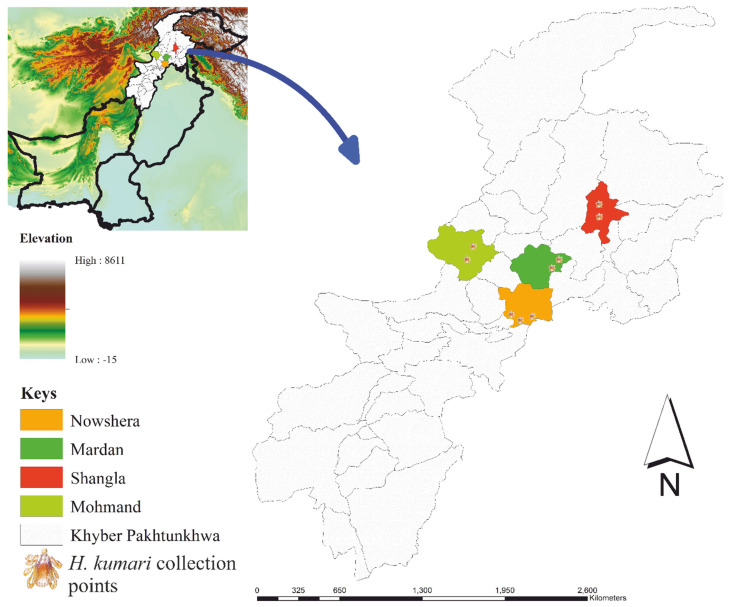
Elevation based map showing collection points of *Hyalomma kumari* in north of Pakistan.

Ticks were collected from herds of grazing goats and sheep in four districts including Nowshera (34.0105° N, 71.9876° E), Mardan (34.1986° N, 72.0404° E), Shangla (34.8872° N, 72.7570° E), and Mohmand (35.2227° N, 72.4258° E), Pakistan. Nowshera district consists mainly of sandy plains surrounded by low elevated hills, comprised of a semi-arid zone. The district Mardan can be divided into two parts: a hilly area in the northeast and a plain area in the southeast. The hills run the length of the district’s northern border. The southwestern part is largely made up of fertile plains with minor scattered hills. District Shangla comprises small valleys surrounded by forest covered high mountains. Natural geographic borders delineate the district Swat, which is centered on the Swat River. Mountains surround the valley on all sides, the high valleys and alpine meadows of Swat-Kohistan, a place where several glaciers originated, are the northernmost area of Swat district. Coordinates of collecting sites in each district were retrieved from Google Map and spread on a Microsoft Excel sheet to design a map using ArcGIS 10.3.1 (Figure 7).

### 4.2. Collection, Preservation and Morphological Identification

Ticks were collected manually from 20 grazing herds of goats and sheep in the study region. Among them, 163 hosts (86 goats, 77 sheep) were infested out of 295 examined hosts (164 goats and 131 sheep). The selected districts where hosts were examined included Mohmand 48/85 (26/47 goats, 22/38 sheep), Nowshera 43/81 (24/44 goats, 19/37 sheep), Shangla 38/74 (21/40 goats, 17/34 sheep), and Mardan 34/55 (19/31 goats, 15/24 sheep). Collected ticks were put in safe lock Eppendorf tubes containing 100% ethanol, which were labeled with information regarding host type, gender, and area of collection. Before identification, ticks were cleaned in order to compare and clarify each taxonomic character with previous standard identification keys [12,33]. After morphological identification, only *H. kumari* ticks were considered for further analysis.

### 4.3. DNA Extraction and Polymerase Chain Reaction (PCR)

Prior to the DNA extraction, the ticks were washed with distilled water and 70% ethanol to remove the contaminants and dried on sterile filter paper. Onward, these ticks were ground in the sterile Eppendorf using scalps and scissors. Whole genomic DNA was extracted manually from 83 randomly selected ticks (unfed) by using the phenol-chloroform method (Table 1) [56]. The purity and quantity of the extracted genomic DNA was checked by a NanoDrop (NanoQ, Optizen, Korea). For the genetic characterization of the *H. kumari*, partial fragments of the tick mitochondrial cytochrome c oxidase subunit 1 (*cox I*) and 16S rRNA genes, and tick nuclear second internal transcribed spacer (ITS-2) were amplified using conventional PCR assays Table 2. PCR reagents were prepared in a 25 µL containing 2 µL genomic DNA (60 ng), 8.5 µL nuclease free water, 1 µL each forward and reverse primer (10 µM), and 12.5 µL DreamTaq green 2x PCR MasterMix (Thermo Scientific, Waltham, MA, USA). *Hyalomma kumari*-associated Rickettsia was targeted and characterized through the amplification of partial sequences of the rickettsial citrate synthase (*gltA*), outer membrane protein A (*ompA*), and outer membrane protein B (*ompB*) genes (Table 1) using the aforementioned reagents mixture. *Hyalomma anatolicum* DNA and *Rickettsia massiliae* DNA were taken as positive controls, while PCR water as a negative control. The amplified PCR products were run on 1.8% agarose gel electrophoresis and visualized through Gel Documentation (LGD-101, Labocon, Hampshire, UK). The resultant amplicons were cleaned and purified through a DNA purification Kit (Invitrogen™JetFlex™, Invitrogen, Waltham, MA, USA).

**Table 2:** Primers used to amplify partial sequences of tick and rickettsial genes.

Organisms and Genes	Primers Sequences (5′-3′)	Amplicon Size (bp)	Annealing Temperature	Reference
Ticks				
16S rRNA	F-CCGGTCTGAACTCAG R-CAATGATTTTTTAAA	460	54 °C	[36]
*cox I*	F-GGAACAATATATTTAATTTTTGG R 5-ATCTATCCCTACTGTAAATATATG	801	55 °C	[46]
ITS-2	F-CCATCGATGTGAAYTGCAGGACA R-GTGAATTCTATGCTTAAATTCAGGGGGT	900	55 °C	[57]
*Rickettsia*				
*gltA*	F-GCAAGTATCGGTGAGGATGTAAT R-GCTTCCTAAAATTCAATAAATCAGGAT	401	56 °C	[58]
*ompA*	F-ATGGCGAATATTTCTCCAAAA R-GTTCCGTTAATGGCAGCATCT	532	55 °C	[59]
*ompB*	F-CCGCAGGGTTGGTAACTGC R-CCTTTTAGATTACCGCCTAA	862	50 °C	[60]

### 4.4. Sequencing and Phylogenetic Analyses

Twelve amplicons, four of each 16S rRNA, *cox I,* and ITS-2, and portions of all amplified rickettsial genes (*gltA*, *ompA*, *ompB*) of the positive samples mentioned in Table 1, were sequenced bi-directionally (Macrogen, Seoul, Republic of Korea). The obtained sequences were assembled and trimmed to eliminate the poor nucleotide regions using SeqMan V. 5 (DNASTAR, Madison, WI, USA). Trimmed sequences were subjected to BLAST (Basic Local Alignment Search Tool) at NCBI (National Center for Biotechnological Information) to download identical sequences [61]. Downloaded sequences, along with obtained sequences, and out group were aligned using ClustalW multiple alignment in BioEdit V.7.0.5 [62]. Aligned sequences were used to construct phylogenetic trees through the Tamura-Nei model and maximum-likelihood statistical method, keeping bootstrap value 1000 in MEGA-11 [63].

### 4.5. Statistical Analysis

Microsoft Excel V.2016 (Microsoft 365^®^) was used for the descriptive analyses of the obtained data. Chi-square multiple variance test was accomplished in the GraphPad Prism version 5, considering *p*-value < 0.05 as significant. This test was carried out to find the significance of the prescribed data as well as to find the difference between variables. The tested variables were male, female and nymphs infesting different genders of the reported hosts [22].

## 5. Conclusions

This is the first extensive analysis based on the prevalence of *H. kumari* in the KP, Pakistan. Furthermore, *R. conorii* detection in *H. kumari* represents the first report of R. conorii in this tick. Further studies should be evaluated regarding the prevalence, genetic characterization of *H. kumari* and their associated *Rickettsia*.

## Figures and Tables

**Table 1:** Number of *Hyalomma kumari* ticks according to geographic districts of Pakistan, host species, and gender, and the results of these ticks for detection of *Rickettsia conorii* DNA by molecular analyses.

Districts	Hosts	Host Gender	No. of Ticks	Tick Life Stages			Molecularly Analyzed Ticks	*Rickettsia* Positive (%)	*p*-Value *
				Female	Male	Nymph			
Nowshera	Goat	male	27	13	10	4	11 F, 9 M, 4 N	1 F, 1 M, 1 N (12.5)	0.0058
		female	39	20	13	6			
	Sheep	male	23	11	9	3			
		female	29	14	12	3			
Mardan	Goat	male	20	10	8	2	8 F, 6 M, 5 N	1 F, 1 N (10.5)	
		female	24	12	11	1			
	Sheep	male	15	8	6	1			
		female	21	11	8	2			
Shangla	Goat	male	22	13	6	3	10 F, 5 M, 3 N	1 M (5.5)	
		female	28	16	11	1			
	Sheep	male	18	9	7	2			
		female	23	10	8	5			
Mohmand	Goat	male	29	15	12	2	9 F, 9 M, 4 N	1 F, 1 M (9.1)	
		female	35	14	13	8			
	Sheep	male	25	15	8	2			
		female	31	13	16	2			
**Total**			409	204	158	47	38 F, 29 M, 16 N	3 F, 3 M, 2 N (9.6)	

* Statistic test: chi-square. F: females; M: males; N: nymphs).

## Data Availability

Not applicable.

## Supplementary Materials

The following supporting information can be downloaded at: https://www.mdpi.com/article/10.3390/ani13091488/s1, Figure S1: PCR amplified *cox I* DNA. 1; molecular marker, 2; control negative (PCR water), 3; *Hyalomma kumari cox I*, 4; control positive (*Hyalomma anatolicum cox I*). Figure S2: PCR amplified 16S rDNA. 1; molecular marker, 4; control negative (PCR water), 2-3,5-7; *Hyalomma kumari* 16S rDNA, 8; control positive (*Hyalomma anatolicum* 16S rDNA). Figure S3: PCR amplified ITS-2 DNA. 1; molecular marker, 5; control negative (PCR water), 2-3; *Hyalomma kumari* ITS-2 DNA, 4; control positive (*Hyalomma anatolicum* ITS-2). Figure S4: PCR amplified Rickettsial *ompA*. 1; molecular marker, 4; control negative (PCR water), 2; *Rickettsia conorii ompA*, 3; control positive (*Rickettsia massiliae ompA*). Figure S5: PCR amplified Rickettsial *ompB*. 1; molecular marker, 4; control negative (PCR water), 2-3,5-6; *Rickettsia conorii ompB*, 7; control positive (*Rickettsia massiliae ompB*). Figure S6: PCR amplified Rickettsial *gltA*. 1; molecular marker, 4; control negative (PCR water), 2; *Rickettsia conorii gltA*, 3; control positive (*Rickettsia massiliae gltA*).
