# Supplementary material for: First Report of Rickettsia conorii in Hyalomma kumari Ticks
Source: Animals (Basel). 2023 Apr 27;13(9):1488. doi: 10.3390/ani13091488 (PMC10177544; doi:10.3390/ani13091488)
Supplement: Supplementary file 1 [file animals-13-01488-s001.zip › animals-2224169-supplementary.pdf]

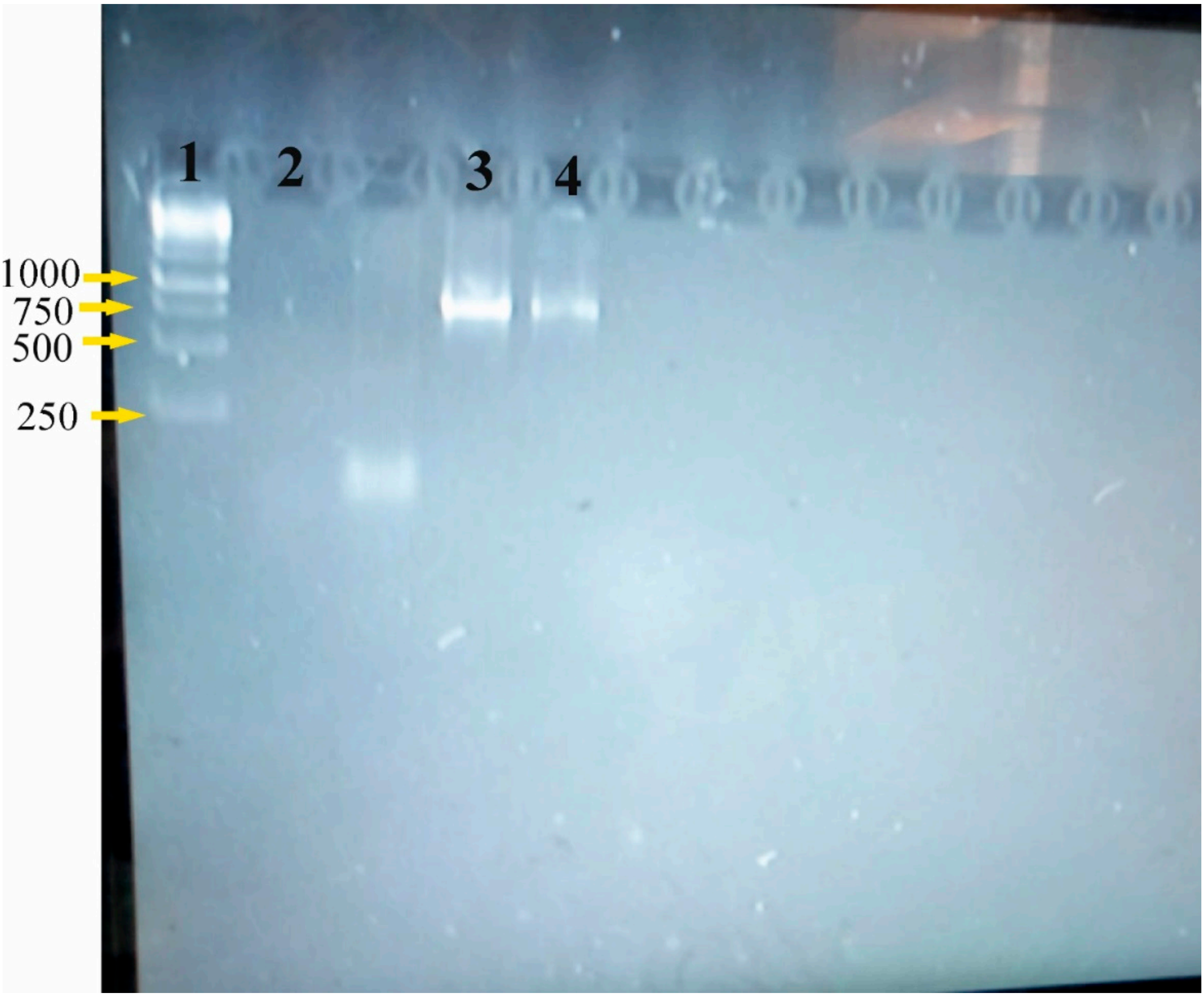

Figure S1. PCR amplified *cox I* DNA. 1; molecular marker, 2; control negative (PCR water), 3; *Hyalomma kumari cox I*, 4; control positive (*Hyalomma anatolicum cox I*).

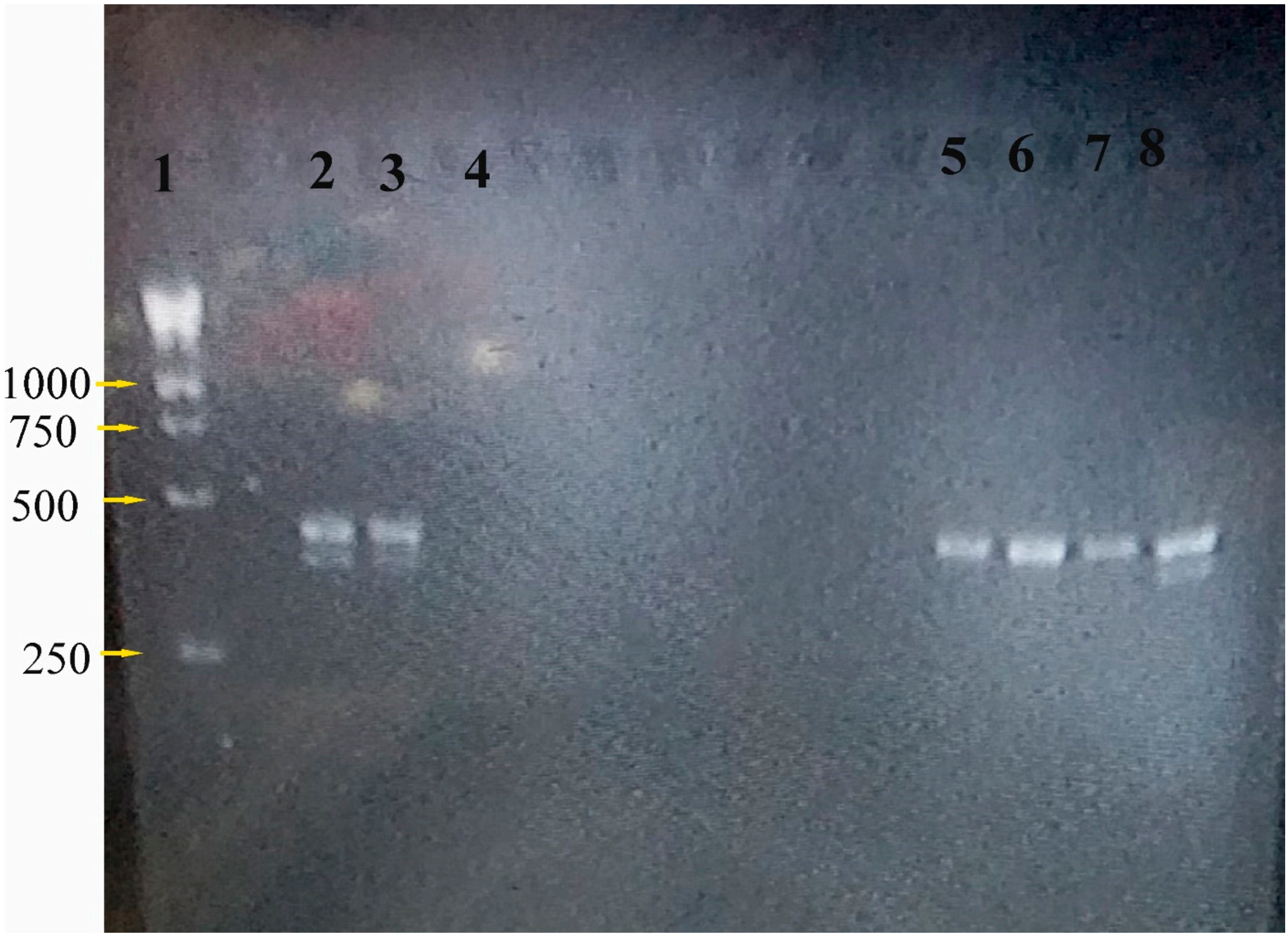

Figure S2. PCR amplified 16S rDNA. 1; molecular marker, 4; control negative (PCR water), 2-3,5-7; *Hyalomma kumari* 16S rDNA, 8; control positive (*Hyalomma anatolicum* 16S rDNA).

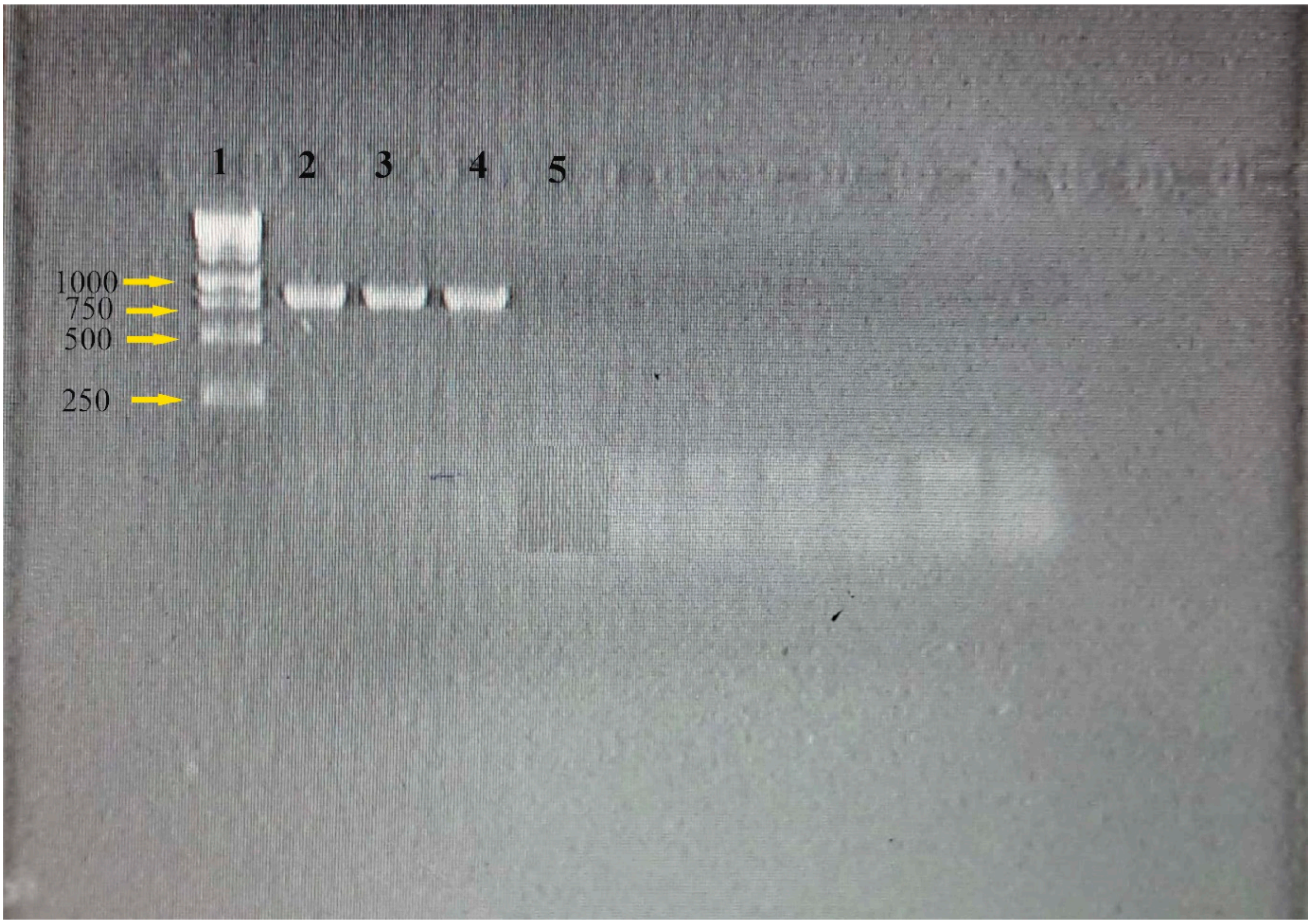

Figure S3. PCR amplified ITS-2 DNA. 1; molecular marker, 5; control negative (PCR water), 2-3; *Hyalomma kumari* ITS-2 DNA, 4; control positive (*Hyalomma anatolicum* ITS-2).

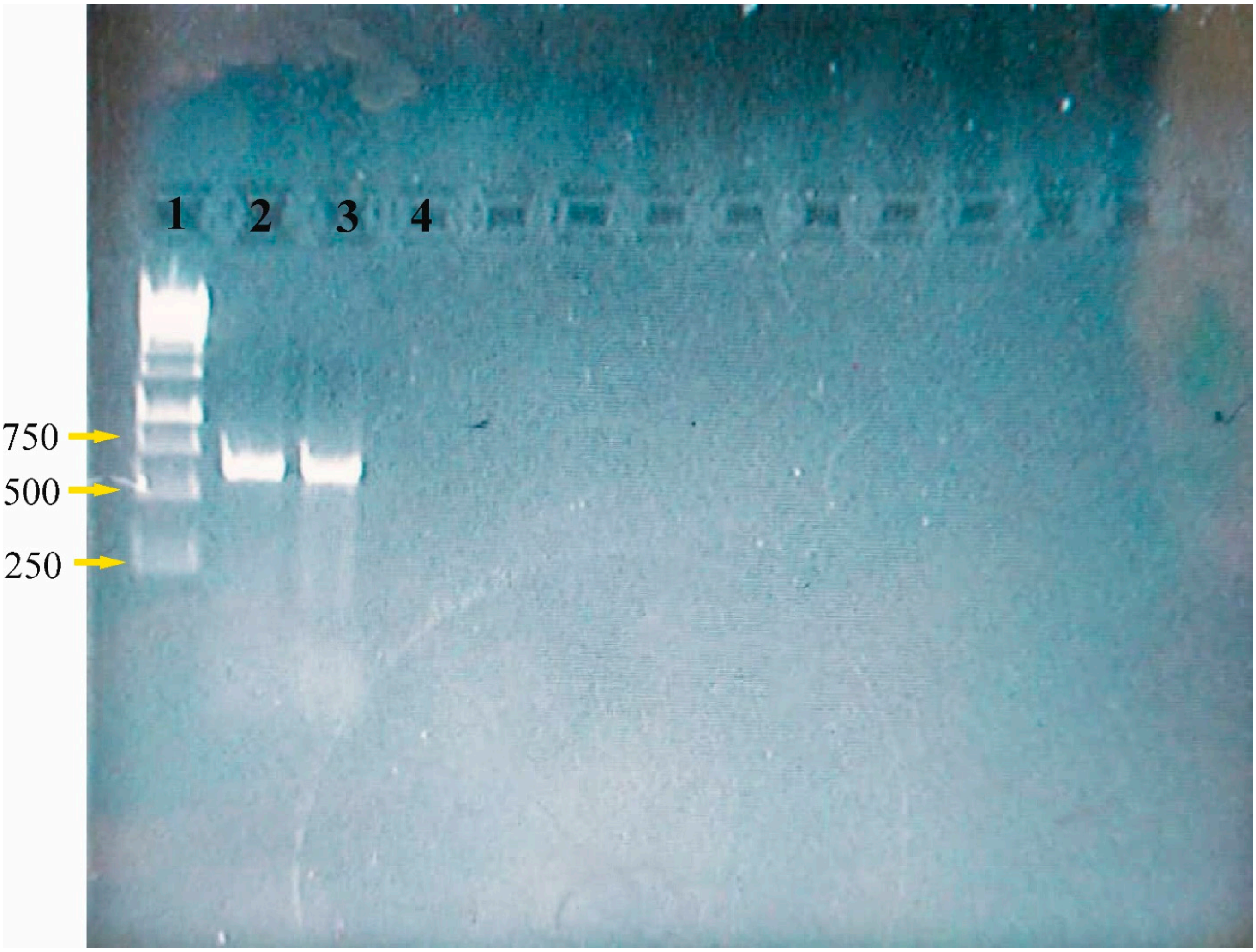

Figure S4. PCR amplified Rickettsial *ompA*. 1; molecular marker, 4; control negative (PCR water), 2; *Rickettsia conorii ompA*, 3; control positive (*Rickettsia massiliae ompA*).

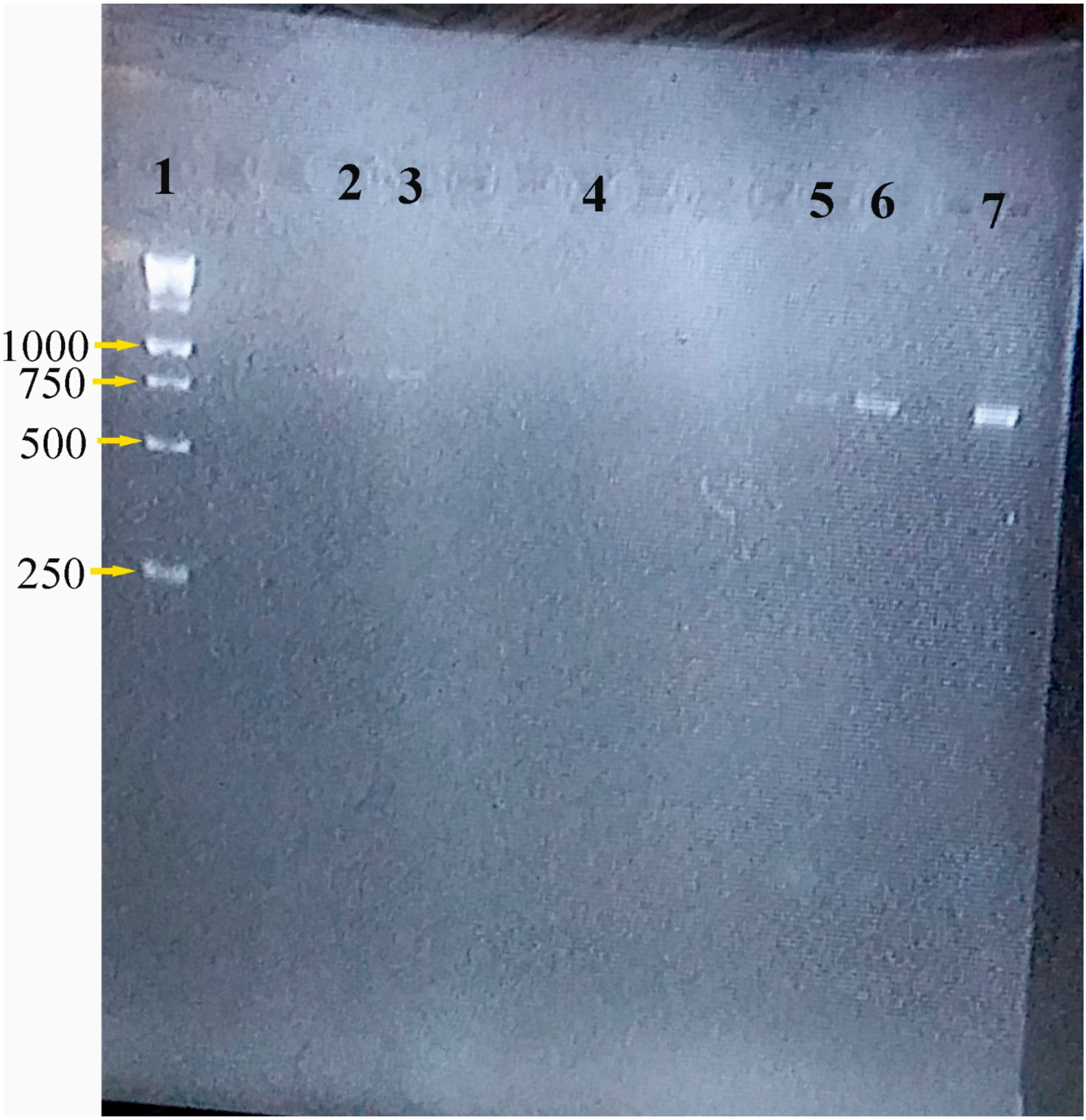

Figure S5. PCR amplified Rickettsial *ompB*. 1; molecular marker, 4; control negative (PCR water), 2-3,5-6; *Rickettsia conorii ompB*, 7; control positive (*Rickettsia massiliae ompB*).

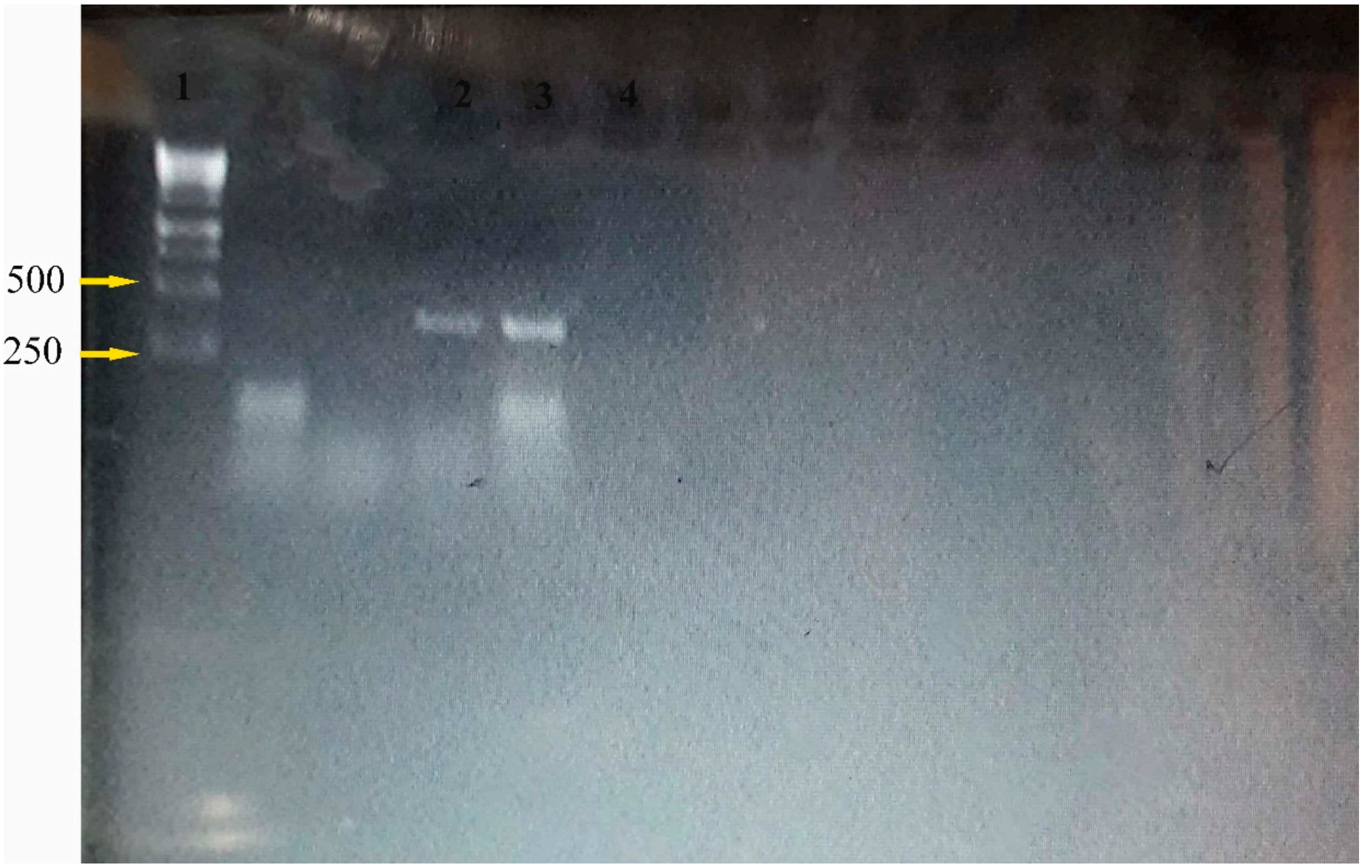

Figure S6. PCR amplified Rickettsial *gltA*. 1; molecular marker, 4; control negative (PCR water), 2; *Rickettsia conorii gltA*, 3; control positive (*Rickettsia massiliae gltA*).
